# The Improved Antigen Uptake and Presentation of Dendritic Cells Using Cell-Penetrating D-octaarginine-Linked PNVA-co-AA as a Novel Dendritic Cell-Based Vaccine

**DOI:** 10.3390/ijms25115997

**Published:** 2024-05-30

**Authors:** Yuri Fujioka, Hideto Ueki, Ruhan A, Akari Sasajima, Takumi Tomono, Masami Ukawa, Haruya Yagi, Shinji Sakuma, Koichi Kitagawa, Toshiro Shirakawa

**Affiliations:** 1 Department of Advanced Medical Science, Graduate School of Science, Technology and Innovation, Kobe University, Kobe 650-0017, Japan; yuriarbachakov91@gmail.com (Y.F.); far.e.is.w@gmail.com (H.U.); aruhan627@yahoo.co.jp (R.A.); akss.araan2@gmail.com (A.S.); ko1.kitagawa@gmail.com (K.K.); 2 Department of Urology, Graduate School of Medicine, Kobe University, Kobe 650-0017, Japan; 3 Faculty of Pharmaceutical Sciences, Setsunan University, Osaka 573-0101, Japan; takumi.tomono@pharm.setsunan.ac.jp (T.T.); masami.ukawa@pharm.setsunan.ac.jp (M.U.); haruya.yagi@setsunan.ac.jp (H.Y.); sakuma@pharm.setsunan.ac.jp (S.S.)

**Keywords:** cancer immunotherapy, dendritic cell vaccine, cell-penetrating polymer, drug delivery system

## Abstract

Cancer immunotherapy using antigen-pulsed dendritic cells can induce strong cellular immune responses by priming cytotoxic T lymphocytes. In this study, we pulsed tumor cell lysates with VP-R8, a cell-penetrating D-octaarginine-linked co-polymer of N-vinylacetamide and acrylic acid (PNVA-co-AA), into the DC2.4 murine dendritic cell line to improve antigen uptake and then determined the anti-tumor effect in tumor-bearing mice. DC2.4 cells were pulsed with the cell lysate of EL4, a murine lymphoma cell line, and VP-R8 to generate the DC2.4 vaccine. For the in vivo study, DC2.4 cells pulsed with EL4 lysate and VP-R8 were subcutaneously injected into the inguinal lymph node to investigate the anti-tumor effect against EL4 and EL4-specific T cell immune responses. VP-R8 significantly improved antigen uptake into DC2.4 compared to conventional keyhole limpet hemocyanin (*p* < 0.05). The expression of MHC class I, MHC class II, and CD86 in DC2.4 cells significantly increased after pulsing tumor lysates with VP-R8 compared to other treatments (*p* < 0.05). The intra-lymph node injection of DC2.4 pulsed with both VP-R8 and EL4 lysate significantly decreased tumor growth compared to DC2.4 pulsed with KLH and lysates (*p* < 0.05) and induced tumor-infiltrating CD8T cells. The DC2.4 vaccine also remarkably increased the population of IFN-gamma-producing T cells and CTL activity against EL4 cells. In conclusion, we demonstrated that VP-R8 markedly enhances the efficiency of dendritic cell-based vaccines in priming robust anti-tumor immunity, suggesting its potential as a beneficial additive for dendritic cell-based immunotherapy.

## 1. Introduction

Cancer is one of the most common causes of death in Japan. In 2021, approximately 26.5% of all deaths were attributed to malignancies. Cancer mortality has consistently risen as the top cause of death since 1981. The mortality rate from cancer is predicted to continue to increase annually [1]. The current modalities of cancer treatment include surgery, radiation therapy, and chemotherapy, but each modality presents significant challenges: surgeries are invasive and impose significant burden on patients [2], and both chemotherapy and radiation therapy can damage healthy tissues and are unable to target cancer cells specifically [3]. In light of these issues, cancer immunotherapy, which utilizes the patient’s own immune system to kill cancer cells, has been gaining attention as a novel and important approach [4].

Cancer immunotherapy harnesses the patient’s immune system to induce an immune response against cancer cells. Immunotherapies, particularly immune checkpoint inhibitors for solid tumors and Hodgkin’s lymphoma, as well as adoptive immunotherapy, are being actively pursued [5]. However, most immunotherapies, other than immune checkpoint inhibitors, have not proven to be effective as standalone treatments for advanced solid tumors. Research efforts worldwide are focused on improving therapeutic outcomes [6]. One of these efforts involves dendritic cell-based vaccines.

Dendritic cell-based vaccines leverage the powerful antigen-presenting capabilities of dendritic cells, which play a crucial role in cancer immune responses. These cells present fragments of cancer antigens on their cell membrane and convey the characteristics of cancer cells to lymphocytes such as cancer antigen-specific cytotoxic T lymphocytes [CTL] and CD4T cells via major histocompatibility complex (MHC) classes I and II and co-stimulatory molecules CD80 and CD86, thus activating T cells [7]. Dendritic cell-based vaccines involve ex vivo methods where cancer antigens, such as tumor lysates, are introduced into a patient’s dendritic cells, which are then cultured and reintroduced into the patient’s body. This process enables dendritic cells to present cancer antigens and activate cancer CTLs, leading to a specific cancer cell cytotoxicity [7]. Sipuleucel-T, a dendritic cell-based vaccine presenting prostatic acid phosphatase (PAP), has been approved in the United States for metastatic castration-resistant cancer. However, the therapeutic effect was limited and did not significantly extend patient survival, approximately 4.1 months compared to untreated patients [8,9]. One possible reason for these limited therapeutic effects could be the inadequate uptake of cancer antigens by dendritic cells. Improving the efficiency of antigen uptake by dendritic cells may enhance the therapeutic effects of dendritic cell therapy [10]. Several drug delivery materials such as liposome, keyhole limpet hemocyanin, or nanoparticles including Poly(D,L-lactic-co-glycolic acid) (PLGA) have been reported to improve antigen uptake and presentation in dendritic cells, but the feasibility is still controversial [11,12,13]. Moreover, the type of antigen loading into dendritic cells should also be improved. The limitations of peptide loading can be overcome by administering whole protein for dendritic cells to uptake and process. The advantage of administering whole protein is that after dendritic cell processing, multiple peptides are available that bind both MHC class I and II and multiple HLA types [14]. Our research focuses on a D-octaarginine-linked polymer that enhances cellular membrane penetration.

Recently, we developed the co-polymer of N-vinylacetamide and acrylic acid (PNVA-co-AA) by grafting D-octaarginine onto its backbone to improve antigen uptake and presentation in dendritic cells by the induction of macropinocytosis [15]. VP-R8, one of the candidates of the D-oligoarginine-linked polymer, induces macropinocytosis by electrostatically interacting with the negatively charged cell membrane, thereby facilitating the uptake of positively charged molecules into the cell [16]. In this process, low-membrane permeability molecules near the oligoarginine are incidentally taken into the cell. Due to competition between macropinosomes, the oligoarginine-fixed polymer itself is not taken up by the cell. Instead, it remains membranous, repeatedly inducing macropinocytosis and significantly enhancing the cellular uptake of low-membrane permeability molecules [15]. By mixing VP-R8 with tumor-associated antigens and adding it to dendritic cells, the cells incidentally take up nearby tumor-associated antigens, which are low-membrane permeability molecules.

The objective of this study is to develop a dendritic cell-based vaccine therapy using VP-R8, with malignant lymphoma selected as the model. Consequently, we assessed the anti-tumor effects of administering dendritic cell-based vaccines that incorporated cancer antigens using VP-R8, along with the induction effects on tumor-infiltrating lymphocytes and cellular immune responses. These were evaluated using the mouse-derived malignant lymphoma cell line EL4 in a mouse model.

## 2. Results

### 2.1. Antigen Uptake of DC2.4 with VP-R8

**Figure 1:**
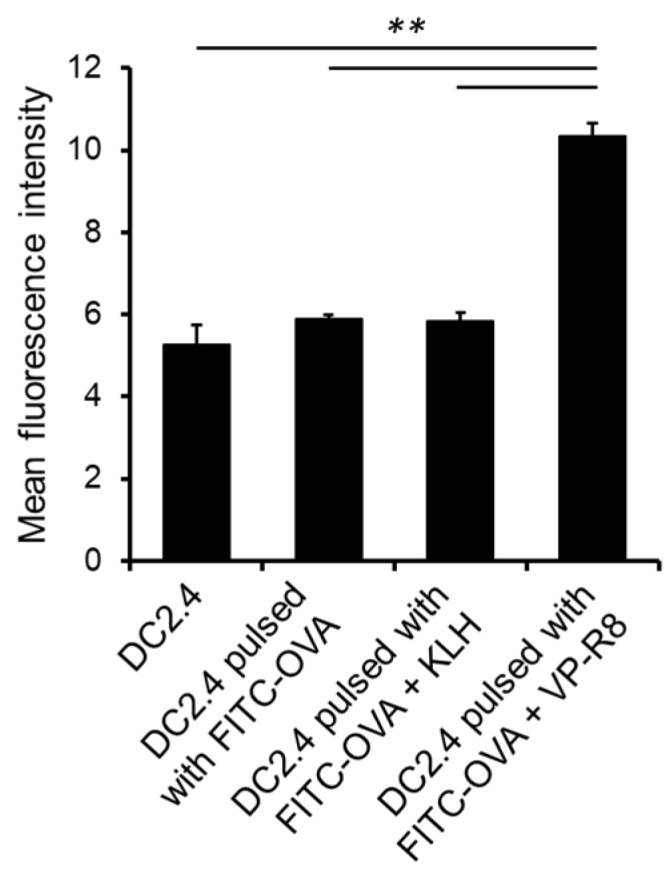
Mean fluorescence intensity (MFI) of FITC-ovalbumin (OVA) in DC2.4 cells. DC2.4 cells were incubated for 1 h with OVA/mixture of OVA and KLH/ mixture of OVA and VP-R8; then, fluorescence was analyzed by flow cytometry. Mean fluorescence intensity (MFI) of FITC-OVA in DC2.4 cells was significantly higher after treatment with VP-R8 compared to keyhole limpet hemocyanin (KLH) (** *p* < 0.01) in vitro, indicating that VP-R8 improved antigen uptake in DC2.4.

A significant increase in mean fluorescence intensity (MFI) was observed in DC2.4 pulsed with the mixture of OVA and VP-R8, compared to another treatment group. The average MFI values for each group were 5.27, 5.89, 5.84, and 10.3, respectively. The Standard deviation (SD) for each group was 0.82, 0.19, 0.35, and 0.55, respectively. These results indicated that VP-R8 significantly enhances the antigen uptake capability of DC2.4 (*n* = 3, *p* < 0.01, partial η^2^ = 0.96) (Figure 1).

### 2.2. Viability after Uptake of EL4 Lysate into DC2.4 Cells

**Figure 2:**
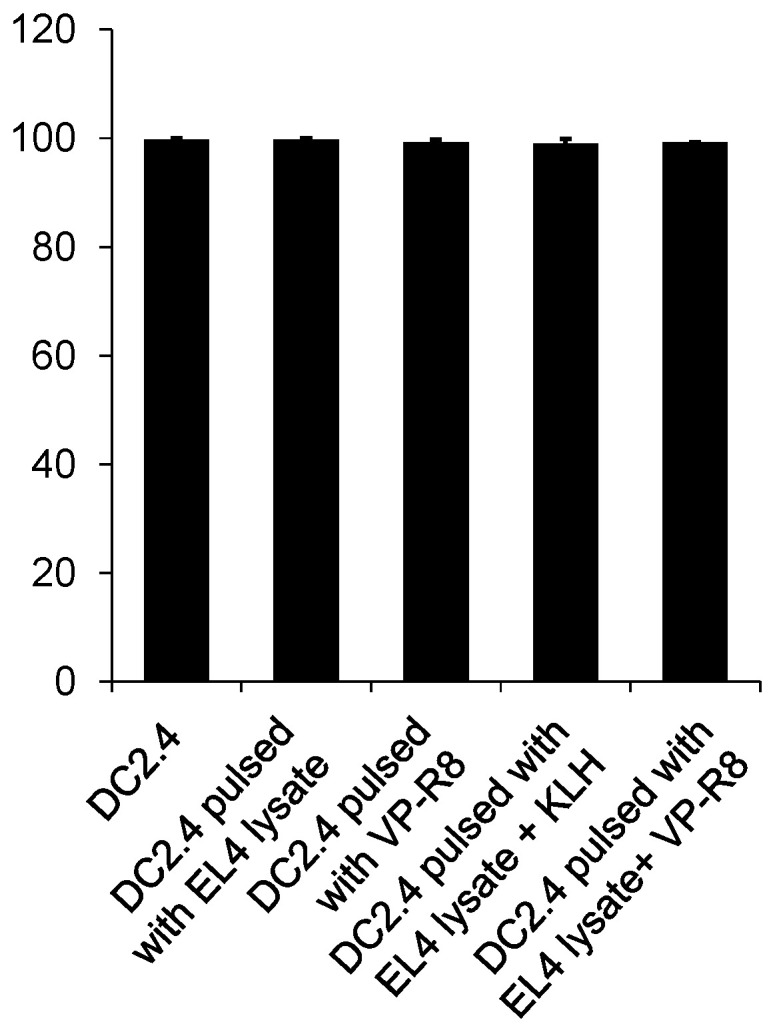
Viability of DC2.4 cells treated with EL4 lysate, and VP-R8. DC2.4 cells were cultured for 1 day after pulsation with EL4 lysate and VP-R8. There was no significant change in viability after antigen uptake in vitro.

After incubation with EL4 lysate and VP-R8 for 1 h, the viability of DC2.4 was determined by flow cytometry. The average viability percentages for each group were 99.80, 99.77, 99.22, 99.00, and 99.20%, respectively. The SDs for each group were 0.34, 0.39, 0.89, 1.54, and 0.14, respectively. There was no significant change in viability between five kinds of treatment groups (*n* = 3, *p* > 0.05, partial η^2^ = 0.19) (Figure 2). These results indicated that pulsing with EL4 lysate with VP-R8 was not toxic for DC2.4.

### 2.3. Evaluation of Surface Molecules on DC2.4 after Antigen Uptake with VP-R8

After the uptake of EL4 lysate with VP-R8, we measured the expression of surface molecules, including MHC class I, MHC class II, CD80, and CD86 on DC2.4 cells, using flow cytometry. The results showed the following averages and SDs for each molecule across the groups:

MHC class I: Average expressions were 366.57, 430.39, 428.56, 402.56, and 473.55 with SDs of 59.13, 16.30, 19.85, 46.09, and 21.11, respectively.

MHC class II: Average expressions were 156.00, 175.15, 138.77, 178.02, and 191.64 with SDs of 8.96, 10.71, 1.03, 12.90, and 4.15, respectively.

CD80: Average expressions were 198.30, 237.61, 292.90, 236.44, and 277.79 with SDs of 34.92, 7.58, 13.72, 16.61, and 2.89, respectively.

CD86: Average expressions were 161.02, 181.13, 199.62, 190.24, and 220.88 with SDs of 26.11, 16.14, 11.07, 8.59, and 13.39, respectively.

**Figure 3:**
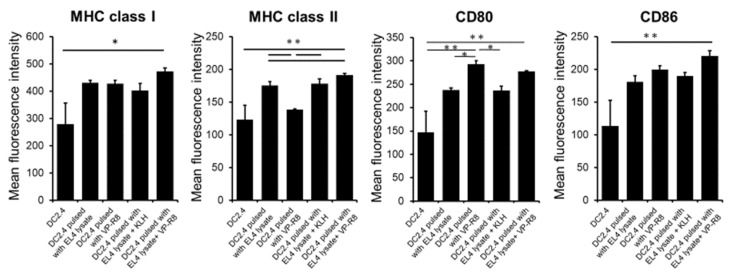
The expression of MHC class I, MHC class II, CD80, and CD86 in DC2.4 cells. The pulsation of EL4 tumor lysate with VP-R8 significantly increased the expression of MHC class I, MHC class II, and CD86 in DC2.4 compared to non-pulse treatment in vitro (* *p* < 0.05, ** *p* < 0.01).

The expression of MHC class I (*p* < 0.05, partial η^2^ = 0.58), MHC class II (*p* < 0.01, partial η^2^ = 0.87), CD80 (*p* < 0.01, partial η^2^ = 0.83), and CD86 (*p* < 0.01, partial η^2^ = 0.69) significantly increased with the pulsation of EL4 tumor lysate with VP-R8 compared to non-pulse treatment in vitro (Figure 3).

### 2.4. In Vivo Anti-Tumor Effect of DC2.4 Vaccine Pulsed with EL4 Lysate and VP-R8

**Figure 4:**
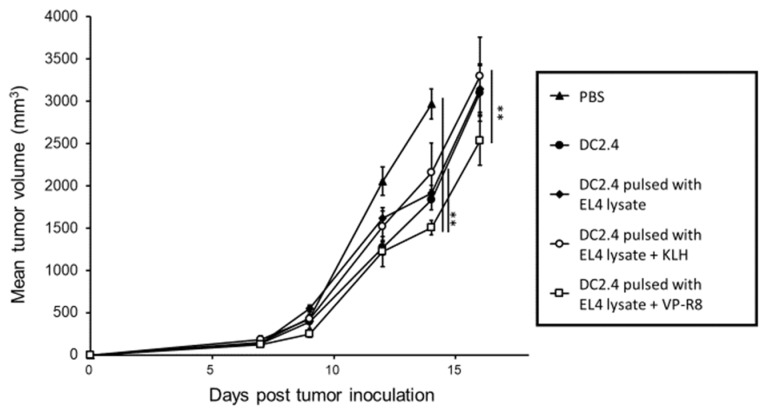
Anti-tumor effect of dendritic cell-based vaccine in mice. Intra-lymph node injection of DC2.4 pulsed with both EL4 lysate and VP-R8 significantly decreased tumor growth compared to DC2.4 pulsed with both EL4 lysate and KLH (** *p* < 0.01), and PBS control (** *p* < 0.01) after tumor inoculation (*n* = 5).

After in vivo tumor transplantation, the intra-lymph node injection of DC2.4 pulsed with EL4 lysate and VP-R8 was conducted three times. After treatment, vaccination significantly decreased tumor volume compared to DC2.4 pulsed with both EL4 lysate and KLH (*p* < 0.01, partial η^2^ = 0.78 in days 14) (*p* < 0.01, partial η^2^ = 0.57 in days 16) and PBS control (*p* < 0.01, partial η^2^ = 0.78 in days 14) (Figure 4). As for day 14, the average tumor volumes for each group starting from PBS were 2967.66, 1835.50, 1916.21, 2159.27, and 1506.19 mm^3^ with SDs of 391.71, 262.12, 205.21, 345.22, and 191.56, respectively. As for day 16, the averages for each group starting from PBS were 4339.21, 3102.16, 3144.15, 3300.25, and 2533.27 mm^3^, with SDs of 704.86, 339.76, 615.52, 452.36, and 653.07, respectively. There were no serious adverse effects during treatment.

### 2.5. Tumor-Infiltrating T Cells after Vaccination with DC2.4 Pulsed with EL4 Lysate and VP-R8

**Figure 5:**
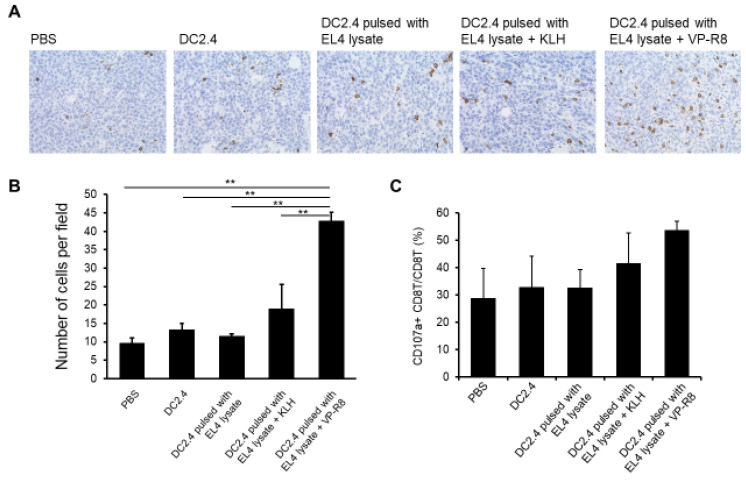
Tumor-infiltrating CD8T cells after vaccination with DC2.4 pulsed with EL4 lysate and VP-R8. After the last vaccination, tumors were isolated, and tumor-infiltrating CD8T cells were observed under a microscope at magnification ×400 (**A**). The average CD8 lymphocyte counts per field of view were determined and statistically analyzed (** *p* < 0.01) (**B**). The proportion of CD107a-positive CD8T cells in EL4 tumors after vaccination was determined by flow cytometry (**C**) (*n* = 5).

Immunohistochemical staining showed that the number of tumor-infiltrating CD8T cells was significantly increased in EL4 tumors in mice treated with DC2.4 pulsed with EL4 lysate and VP-R8 compared to other treatment groups (*p* < 0.01, partial η^2^ = 0.63) (Figure 5A,B). The average number of cells per field for each group was 9.67, 13.22, 11.56, 18.89, and 42.78. The SD for each group was 6.30, 5.80, 7.73, 4.89, and 18.23. Flow cytometry analysis showed a higher population of CD107a-positive CD8T cells in the EL4 tumors after treatment with DC2.4 pulsed with EL4 lysate and VP-R8 compared to control DC2.4 treatment (*n* = 3 *p* > 0.05, partial η^2^ = 0.32) (Figure 5C). The average proportion of CD107a-positive CD8T cells for each group was 28.76, 32.82, 32.50, 41.48, and 53.61. The SD for each group was 19.03, 19.55, 11.71, 19.18, and 5.82. These results indicated that the intra-lymph node injection of DC2.4 pulsed with EL4 lysate and VP-R8 induced tumor-infiltrating CD8T cells with degranulated markers eliciting cytotoxicity in tumor cells.

### 2.6. Intracellular Cytokine Staining in Spleen Cells after Vaccination with DC2.4 Pulsed with EL4 Lysate and VP-R8

**Figure 6:**
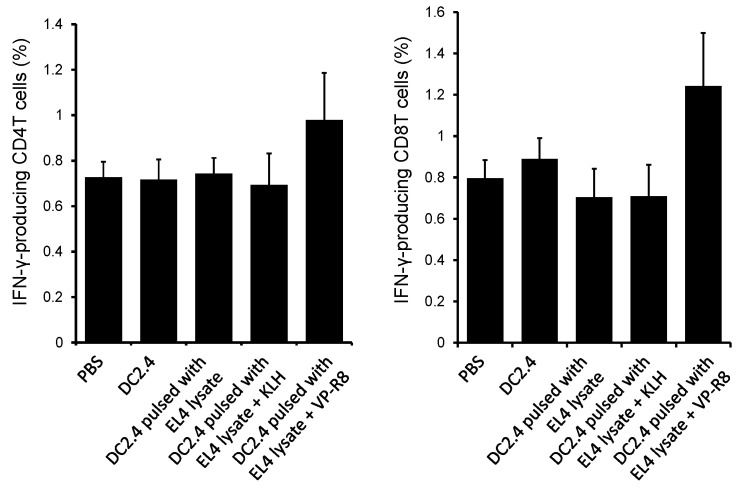
IFN-γ-producing T cells in mouse spleens after vaccination. The population of both CD4T cells and CD8T cells specifically producing IFN-γ was remarkably increased by treatment with DC2.4 pulsed with both EL4 lysate and VP-R8 compared to other treatments (*n* = 5).

After vaccination with DC2.4 pulsed with EL4 lysate and VP-R8, spleen cells were isolated and re-stimulated with mitomycin C-treated EL4 cells to induce cytokine production. The intracellular cytokine staining of spleen cells showed that vaccination with DC2.4 with EL4 lysate VP-R8 substantially increased IFN-γ-producing CD4T cells and CD8T cells compared to control DC2.4 treatment, but the differences were not significant (*p* > 0.05) (Figure 6).

### 2.7. Cytotoxic Activity of CTLs Induced by DC2.4 Vaccine

**Figure 7:**
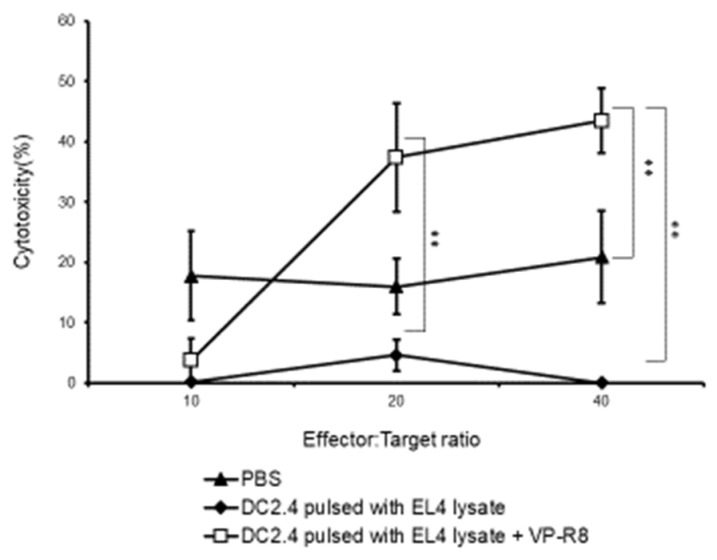
Detection of cytotoxic activities of splenocytes after vaccination with DC2.4 pulsed with EL4 lysate and VP-R8. Splenocytes from mice (*n* = 5) were isolated eight days after last DC2.4 vaccination and re-stimulated with mitomycin-C treated EL4 cells and IL-2 for 6 days in vitro to generate effector cells. These were co-cultured with target cells (EL4) for 6 h at ratios of 40:1, 20:1, and 10:1. Cytotoxicity of effector cells against EL4 cells was assessed (** *p* < 0.01).

After stimulating the splenocytes with EL4 for 6 days, the cytotoxic activity of the splenocytes was assessed in vitro. At the effector/target ratio of 40:1, the mice treated with DC2.4 with EL4 lysate and VP-R8 showed significantly higher cytotoxic activity against EL4 cells compared to the mice treated with DC2.4 with PBS alone and EL4 lysate alone (*p* < 0.01, η^2^ = 0.73) (Figure 7). The average cytotoxicity for each group was 16.64, 0.00, and 43.46, respectively. The SD for each group was 17.15, 0.00, and 12.15, respectively. These results suggested that the intra-lymph node injection of DC2.4 pulsed with EL4 lysate and VP-R8 could induce CTLs against EL4.

## 3. Discussion

Cancer is the primary cause of death in Japan, representing about one-fourth of all deaths in 2020, with its mortality rate rising annually [1]. Among various treatments, cancer immunotherapy, particularly dendritic cell-based vaccines, shows promise as a less invasive and more effective option [17]. Cancer immunotherapy involves processing the patient’s dendritic cells with cancer antigens outside the body and reintroducing them to induce a strong immune response. In terms of utilizing the patient’s own immune system, dendritic cell-based vaccines are a promising candidate for cancer treatment, aiming for efficient antigen presentation and robust immune activation [18]. To introduce the broad range of tumor-associated antigens such as tumor-associated antigens (TAAs) and tumor-specific antigens (TAAs) into dendritic cells, tumor lysates can enhance the maturation and antigen presentation of dendritic cells and induce high anti-tumor immune responses by antigen-specific CTLs, which may prevent tolerance to single-peptide-specific immune responses [19,20]. In this study, we focused on VP-R8, an oligoarginine-linked polymer that facilitates cell membrane penetration, to enhance the antigen uptake and therapeutic effect of dendritic cell-based vaccines. The most common adult hematologic malignancy is lymphoma, including B-cell and T-cell lymphomas. While the survival rate for patients with B-cell lymphoma has significantly improved with the molecular-targeted drug Rituximab, the prognosis for T-cell lymphoma remains poor due to frequent treatment resistance and relapse [21]. Therefore, this study evaluated the anti-tumor effect of a dendritic cell-based vaccine using EL4, a T-cell lymphoma cell line, as an experimental model.

We first showed that pulsing with a mixture of FITC-conjugated OVA and VP-R8 significantly enhanced the antigen uptake of DC2.4 compared to other treatments in vitro. In addition, the mixture of EL4 lysate with VP-R8 did not affect cell viability after pulsation. Electroporation is one of the efficient methods to transduce proteins into dendritic cells to induce dendritic cell-based vaccines, but preserving cell viability after transduction is challenging [22]. Our results suggested that VP-R8 is a safer and more efficient modality to deliver tumor antigens into dendritic cells than conventional electroporation.

Next, we showed that the mixture of EL4 lysate and VP-R8 significantly increased the expression of maturation markers MHC class I, MHC class II, CD80, and CD86 in DC2.4 cells compared to other treatments. Hatfield et al. reported that freeze–thaw tumor cell lysates inhibited the maturation and function of dendritic cells and prevented the upregulation of CD40, CD86, and major histocompatibility complex class II [23]. However, we showed that the mixture of VP-R8 and tumor cell lysate increased the maturation markers of dendritic cells, suggesting that VP-R8 upregulated cell maturation and antigen presentation as well as cell membrane penetration.

In an in vivo mouse model bearing EL4 tumors, DC2.4 loaded with tumor lysate and VP-R8 significantly suppressed tumor growth compared to other treatments. In addition, tumor-infiltrating CD8T lymphocytes significantly clustered in the tumor tissues after vaccination with DC2.4 pulsed with EL4 lysate and VP-R8 compared to other treatments. Immunohistochemical staining implied that the anti-tumor effect may be mediated by CD107a-positive CD8T cells, but the increases in the number of CD107a-positive CD8T cells were not significant. CD107a is a degranulation marker expressed during the degranulation response of CD8T cells, suggesting that the intra-lymph node administration of DC2.4 pulsed with tumor lysate and VP-R8 could enhance the membrane permeability of tumor antigens and induce more activated CD8T cells in the tumor to eliminate tumor cells. We also showed that the DC2.4 vaccine increased the proportion of IFN-γ-producing CD4T and CD8T cells in mouse spleens, but the differences were not significant. This fact may indicate that DC2.4 presented tumor antigens to CD4T cells and CD8T cells and induced tumor antigen-specific T cell immune responses to kill tumor cells. We also showed that the intra-lymph node administration of DC2.4 pulsed with tumor lysate and VP-R8 induced significantly strong CTL activity against EL4 cells after vaccination in mice. Recently, Teng et al. reported that the blockade of the programmed death ligand 1 (PD-L1) immune checkpoint pathway significantly enhanced the anti-tumor immune responses in hepatocellular carcinoma in mice and induced stronger CTL responses [24]. To overcome the weakness in the induction of tumor-specific T cells by our DC2.4 vaccination, this can be improved by the immune checkpoint inhibitor.

The efficiency of antigen uptake and antigen presentation in dendritic cells is one of the greatest challenges for effective dendritic cell-based vaccines. Kim et al. reported Annona muricata L.-derived polysaccharides (ALPs) as an adjuvant candidate that can induce anti-tumor activity [25]. They showed that ALP facilitated phenotypic (surface molecules, cytokines, antigen uptake, and antigen-presenting ability) and functional alterations (T cell proliferation/activation) of DCs in vitro. They also confirmed that the systemic administration of DCs that pulse ALPs and ovalbumin peptides strongly increased CTL activity, the generation of CD107a-positive T cells, and Th1-mediated humoral immunity, with a significant reduction in the tumor growth of murine thymoma. Their results suggested that efficient antigen uptake, antigen presentation, and maturation are key factors for producing an efficient dendritic cell-based vaccine for cancer treatment.

This study has some limitations. First, the dendritic cells and the tumor lysate used in this study were obtained from cultured cell lines and not taken from primary cultures of bone marrow directly harvested from mice. Secondly, we administered the dendritic cell-based vaccine locally, but we should further validate whether the systemic administration of dendritic cells would lead to the same results. Third, some kinds of molecules that are over-expressed in several tumors (e.g., versican) and pathways which are over-facilitated in certain tumors (e.g., PD-1/PD-L1, Wnt-1) could possibly ameliorate the effects of our therapeutic approach by exerting their immuno-modulatory effects [26,27,28]. Fourth, it is not clear which types of peptides or proteins among the various tumor antigens contained in the lysate are taken into DC2.4 [29]. How VP-R8 enhanced whole protein uptake needs to be clarified in future experiments. Fifth, the sample size used in this study may be small; therefore, the statistical results do not accurately reflect the obtained results. Despite these limitations, our research model demonstrated the feasibility of using VP-R8 in the production of dendritic cell-based vaccines.

In conclusion, this study showed that VP-R8, an oligoarginine-linked polymer, can enhance antigen uptake and antigen presentation in DC2.4 cells and that a dendritic cell-based vaccine pulsed with tumor cell lysate and VP-R8 could elicit a strong anti-tumor effect and induce tumor-infiltrating CD8T cells in a tumor-bearing mouse model.

## 4. Materials and Methods

### 4.1. Cell Lines

Murine dendritic cell-based line Dendritic Cell 2.4 (DC2.4), derived from C57BL/6 mice, was kindly provided by Dr. Kenneth L. Rock from the University of Massachusetts Medical School. DC2.4 was cultured in Roswell Park Memorial Institute (RPMI)-1640 medium containing 10% Fetal Bovine Serum (FBS) (Sigma-Aldrich, St. Louis, MO, USA), 1% non-essential amino acids (Nacalai Tesque, Kyoto, Japan), 1% sodium pyruvate (Nacalai Tesque, Kyoto, Japan), 100 U/mL penicillin, 100 μg/mL streptomycin (Nacalai Tesque, Kyoto, Japan), and 0.004% 2-Mercaptoethanol (FUJIFILM Wako Pure Chemical Corporation, Tokyo, Japan) unless otherwise mentioned. RPMI-1640 medium including the above components without FBS (FBS(-)-RPMI medium) was used for antigen uptake by DC2.4. EL4, a murine T cell thymoma cell line derived from C57BL/6 mice, was purchased from ATCC (Manassas, VA, USA) and cultured in RPMI-1640 medium supplemented with 10% FBS, 100 U/mL penicillin, and 100 μg/mL streptomycin.

### 4.2. Preparation of Lysate from EL4

The cultured EL4 cells were washed twice with Dulbecco’s Phosphate-Buffered Saline (D-PBS). The cells were then suspended in D-PBS at 2 × 10⁷ cells/mL and lysed with five freeze–thaw cycles using liquid nitrogen. The supernatant was collected by centrifugation at 10,000 rpm for 10 min at 4 °C.

### 4.3. Evaluation of Antigen Uptake of DC2.4 in Presence of VP-R8

DC2.4 cells (1.0 × 10⁵) were seeded in a 24-well plate and cultured overnight. After the removal of the supernatant, FBS (-)-RPMI medium was added. To evaluate the antigen uptake of DC2.4, 10 µg/mL ovalbumin (OVA)-fluorescein conjugate (Merck, Rahway, NJ, USA), mixtures of 10 µg/mL OVA with 50 µg/mL Keyhole Limpet Hemocyanin (KLH) (FUJIFILM Wako Pure Chemical Corporation), and 10 µg/mL OVA with 50 µg/mL of VP-R8 were added to the DC2.4 cells, respectively. The cells were then incubated at 37 °C for 1 h. After culture, DC2.4 cells were harvested and suspended in D-PBS, and their mean fluorescence intensity (MFI) was measured using a Guava easycyte flow cytometer (Luminex, Austin, TX, USA). The experiment was performed in triplicate.

### 4.4. Viability after EL4 Lysate Uptake with VP-R8

DC2.4 cells (1.0 × 10⁵) were seeded in a 24-well plate and cultured overnight. After the removal of the supernatant, FBS(-)-RPMI medium was added. To determine the viability of DC2.4 after antigen uptake with DC2.4, cell lysate extracted from 3.0 × 10⁵ EL4 cells was mixed with 50 µg/mL KLH or 12.5 µg/mL VP-R8, and then the mixture was added to DC2.4 cells. After incubation at 37 °C for 1 h, the supernatant was removed, and RPM-1640 medium was added into the wells, and DC2.4 cells were further cultured for one day. Harvested DC2.4 cells were suspended in D-PBS, and their viability was measured using Guava^®^ ViaCount™ Reagent (Merck). The experiment was performed in triplicate.

### 4.5. Analysis of Surface Markers of DC2.4 after Antigen Uptake with VP-R8

DC2.4 cells (3.0 × 10⁵ cells) were seeded in a 6-well plate and incubated overnight at 37 °C. The supernatant was removed, and FBS(-)-RPMI medium was added into the culture. The DC2.4 cells were then divided into four groups (*n* = 3): 3.0 × 10⁵ EL4 cells/well lysate, 50 µg/mL VP-R8, a mixture of EL4 lysate with VP-R8, or a mixture of EL4 lysate with KLH. After incubation at 37 °C for 1 h, the supernatant was then discarded, and RPMI-1640 medium was added. The cells were further cultured for an additional 4 days. The cells were harvested and incubated with 1 µg of anti-mouse CD16/32 antibody in staining buffer (1% FBS, 0.09% sodium azide in D-PBS) on ice for 20 min. The cells were stained with FITC-anti-mouse H-2Kb/H-2Db antibody (MHC class I), PE-anti-mouse I-Ab (MHC class II) antibody, PerCP-anti-mouse CD86 antibody, and APC-anti-mouse CD80 antibody (BioLegend, San Diego, CA, USA, respectively) in staining buffer on ice in the dark for 30 min, respectively. The cells were washed, and expressions were measured by flow cytometry.

### 4.6. In Vivo Tumor Challenge Model with EL4

A total of 1.0 × 10⁵ EL4 cells were subcutaneously transplanted into the right flanks of 6-week-old female C57BL/6J mice with equal volumes of Matrigel (Corning, Corning, NY, USA).

### 4.7. Preparation and Administration of DC2.4 Vaccine Loaded with VP-R8 and Tumor Lysate

After culturing DC2.4 cells, FBS(-)-RPMI medium was added into the culture. Mixtures of 2.0 × 1.0⁷ cells/mL EL4 lysate from 3.0 × 10⁷ cells of EL4, a mixture of EL4 lysate and 50 µg/mL Keyhole limpet hemocyanin (KLH) (FUJIFILM Wako Pure Chemical Corporation), and a mixture of EL4 lysate with 12.5 µg/mL VP-R8 were added to 1.0 × 10⁷ cells/3 mL of DC2.4, respectively. The mixtures were incubated at 37 °C for 1 h. Following incubation, the cells were washed and suspended in D-PBS to a final concentration of 1.0 × 10⁶ cells/100 µL, creating the DC2.4 vaccine. The prepared DC2.4 vaccine was administered subcutaneously around the right inguinal lymph nodes on days 7, 10, and 13 after tumor transplantation (*n* = 5). For the untreated group, a subcutaneous injection of D-PBS was injected into the inguinal lymph nodes (*n* = 5). Tumor diameters were measured, and tumor volume was calculated using the following formula: tumor volume = (longest diameter) × (shortest diameter)^2^ × 1/2. The animal experiment was approved by the Institutional committee of Kobe University.

### 4.8. Analysis of Tumor-Infiltrating T Cells

After the above treatment, mice were euthanized on day 14, and the tumors were excised (*n* = 5). To analyze tumor-infiltrating T cells, tumors were homogenized with the tumor dissociation kit (Miltenyi Biotec, Bergisch Gladbach, Germany), according to the manufacture’s manual. Single-cell suspensions were treated with red blood cell lysis buffer (Sigma-Aldrich), followed by washing and centrifugation. After centrifugation, cells were treated with debris removal solution (Miltenyi Biotec) to remove dead cells and debris. Subsequently, 1.0 × 10^6^ cells were resuspended in staining buffer and blocked with 10 µg/mL anti-mouse CD16/32 antibody on ice for 15 min. After washing with staining buffer, the cells were stained for 30 min on ice in the dark with 2 µg/mL PerCP-anti-mouse CD3 antibody, APC-anti-mouse CD8 antibody, and PE-anti-mouse CD107a antibody (Biolegend, San Diego, CA, USA) in staining buffer. The cells were washed with staining buffer and analyzed using a Guava easycyte flow cytometer.

### 4.9. Immunohistochemical Staining for Tumor-Infiltrating T Cells

Resected tumors were fixed with 4% paraformaldehyde and blocked with paraffin. After antigen retrieval, tissues were stained with anti-CD8 alpha antibody (Abcam, Cambridge, UK). The immunohistochemical staining was conducted at Pathotec, LLC (Kobe, Japan). The number of CD8T cells in three fields of view per tumor tissue from each mouse was measured using a light microscope, and the average number of CD8+ T cells per field of view was calculated (*n* = 5).

### 4.10. Measurement of CTL Activity Induced by DC2.4 Vaccine

Other mice were assigned to three treatment groups: DC2.4 alone, DC2.4 with added EL4 lysate, and DC2.4 with a mixture of EL4 and VP-R8. These DC2.4 vaccines were prepared as described above. DC2.4 vaccines were administered subcutaneously around the right inguinal lymph nodes on days 0, 3, and 6. For the untreated group, a subcutaneous injection of D-PBS was given into the inguinal lymph nodes.

Eight days after the last vaccination, mice were euthanized, and spleen cells were collected. The spleen cells (3.0 × 10^7^) were seeded in a 6-well plate and co-cultured with mitomycin C-treated EL4 cells (3.0 × 10^6^) at 37 °C in a 5% CO_2_ incubator for 6 days in the presence of 20 ng/mL IL-2 added on days 1 and 3 of the culture to generate CTLs, according to a previous study [30]. After the culture, spleen cells as effector cells were co-cultured with 5.0 × 10^3^ EL4 cells at effector/target ratios of 40:1, 20:1, and 5:1 in 96-well plates at 37 °C in a 5% CO_2_ for 8 h. After incubation, the culture supernatant was collected, and CTL activity was measured using the CytoTox 96^®^ Non-Radioactive Cytotoxicity Assay (Promega, Madison, WI, USA). CTL activity was calculated using the following formula: Cytotoxic activity (%) = (Experimental release − Effector spontaneous release − Target spontaneous release)/(Target maximum release − Target spontaneous release) × 100.

### 4.11. Intracellular Cytokine Staining for Spleen Cells

Isolated spleen cells (2.0 × 10^6^) were cultured with mitomycin C-treated EL4 cells (2.0 × 10^5^) at 37 °C in a 5% CO_2_ incubator for 12 h in vitro (*n* = 5). Spleen cells were incubated with anti-CD16/32 antibody (BioLegend) for 15 min on ice. Cells were then washed and stained with FITC-anti-mouse CD4, APC-anti-mouse CD8, and PerCP-anti-mouse CD3 (BD Biosciences, Franklin Lakes, NJ, USA, respectively) in staining buffer on ice for 30 min. Cells were washed and fixed with BD fixation/permeabilization solution on ice for 20 min. After washing, spleen cells were stained with PE-anti-mouse IFN-γ antibody (BD Biosciences) for 30 min. Cells were then washed, and fluorescence was determined by a Guava easycyte flow cytometer.

### 4.12. Statistical Analysis

The data from each experiment were statistically processed using a one-way ANOVA followed by the Tukey–Kramer method as a post hoc test for comparisons between multiple groups. Differences among experimental groups were considered significant when *p* < 0.05. Statistical analyses were conducted using EZR version 1.64 (Saitama Medical Center, Jichi Medical University, Shimotsuke-shi, Tochigi-ken, Japan), which is a graphical user interface for R (The R Foundation for Statistical Computing, Vienna, Austria). For our one-way ANOVA, we provided the full statistical model results, including the *p*-value and effect size measures such as eta-squared to indicate the magnitude of the observed effects.

When significant differences were detected by ANOVA, post hoc pairwise comparisons were performed using the Tukey–Kramer method, selected for its robustness in handling unequal sample sizes and variances between groups. These comparisons were accompanied by 95% confidence intervals and the exact *p*-values for each comparison, allowing for a clear and transparent interpretation of the data.

To enhance the clarity of our results, we have also included detailed descriptive statistics for each experimental group, such as the mean, SD, and sample size.

## Data Availability

The data that support the findings of this study are available from the corresponding author upon reasonable request.
